# Clinical benefits including the postoperative inflammatory status of robot-assisted distal gastrectomy for gastric cancer compared with laparoscopic distal gastrectomy in patients with obesity

**DOI:** 10.1007/s11701-025-02296-3

**Published:** 2025-04-10

**Authors:** Kazuaki Matsui, Shinichi Sakuramoto, Tasuku Furube, Masatoshi Yoshizawa, Tetsuro Toriumi, Gen Ebara, Yutaka Miyawaki, Hiroshi Sato

**Affiliations:** Department of Gastroenterological Surgery, Saitama Medical University International Medical Center, 1397-1, Yamane, Hidaka, Saitama, 350-1298 Japan

**Keywords:** Robot-assisted gastrectomy, Laparoscopic gastrectomy, Obesity, CRP, Postoperative inflammation, Gastric cancer

## Abstract

This study aimed to clarify the clinical benefits of robot-assisted distal gastrectomy (RDG) versus laparoscopic distal gastrectomy (LDG) in patients with obesity.

The analysis included 89 and 87 patients in LDG and RDG groups, respectively. The associations between body mass index (BMI) and surgical outcomes including postoperative inflammation were compared between LDG and RDG.

Incidences of postoperative complications did not show a significant difference between LDG and RDG. The operating time and blood loss increased with BMI in the LDG group, whereas no such correlation was observed in the RDG group. While BMI and C-reactive protein (CRP) levels on postoperative day (POD) 3 showed a significant correlation in LDG (R = 0.393, *p* < 0.001), RDG did not show a correlation. In patients with BMI ≥ 25 (kg/m^2^), CRP on POD 3 was significantly lower in RDG than in LDG. Multivariate analysis for CRP on POD 3 in patients with BMI ≥ 25 identified RDG and operating time ≥ 360 min as independent associated factors (*B* = − 6.887; *p* = 0.003 and *B* = 6.068; *p* = 0.011).

RDG was indicated to reduce blood loss and suppress the postoperative CRP elevation compared with LDG, particularly in patients with high BMI.

## Introduction

Following advancements in minimally invasive surgery for gastric cancer, the global adoption of robot-assisted gastrectomies (RGs) has increased in recent years [1]. Although laparoscopic gastrectomy (LG) has been standardized for a safe and curative procedure, several studies have demonstrated the additional surgical benefits of RG, which enables more precise techniques. Regarding short-term outcomes, RG has been reported to result in less blood loss than LG in previous studies [2, 3]. Previous reports suggest that postoperative complications did not differ significantly between LG and RG [4, 5]. These reports indicate no significant differences in long-term survival between LG and RG [2, 6]. These findings likely reflect advancements in surgical technique facilitated by RG and the maturity of LG as a procedure. While the surgical benefits of robotic surgery are compelling, a systematic review reported significantly higher costs for RG compared with LG [1]. Identifying scenarios where RG is particularly advantageous is crucial for advancing robotic surgery in the future.

Despite advancements in minimally invasive gastrectomy, performing gastric cancer surgery on patients with obesity presents unique challenges. Notably, postoperative complications have been reported to increase among patients with obesity [7, 8]. Our previous research demonstrated that body mass index (BMI) significantly correlates with postoperative C-reactive protein (CRP), a key indicator of inflammatory status [9]. Furthermore, elevated postoperative inflammatory status has been reported as an independent risk factor for long-term survival [10, 11]. This study hypothesizes that the precision of robotic surgery can suppress postoperative inflammation, even in the obese patients. The aims of this study were to clarify the association between preoperative obesity and surgical outcomes including postoperative inflammation and to investigate the clinical usefulness of RG compared with LG in patients with obesity.

## Materials and methods

### Patients and gastrectomy

Introducing robotic surgery at our institution, RG for gastric cancer has been the standard procedure since 2022. We investigated the clinical advantages of RG by comparing it to LG, which was performed during its period as the standard procedure. We focused on distal gastrectomy (DG) to eliminate procedure variability. We screened gastric cancer patients who underwent laparoscopic distal gastrectomy (LDG) from January to December 2021 and robot-assisted distal gastrectomy (RDG) from October 2022 to November 2024 at Saitama Medical University International Medical Center, Saitama Prefecture. The preparation period for RG implementation as the standard procedure between January 2022 and September 2022 was excluded. The patients who underwent DG with combined resection of organs except cholecystectomy or noncurative resection were excluded. The extent of lymph node dissection was determined in accordance with the Japanese Gastric Cancer Treatment Guidelines [12]. All surgeries were performed by, or under the supervision of surgeons accredited by the Japanese Society of Gastroenterological Surgery and the Endoscopic Surgical Skill Qualification System in Japan [13]. Surgical anesthesia was administered by anesthesiologists. In principle, general anesthesia was administered immediately before surgery and a patient was extubated immediately after gastrectomy on the day of surgery. Similarly, intraoperative fluid management during gastrectomy was continuously performed by anesthesiologists in accordance with the management for minimally invasive surgery. In this study, a patient with obesity was defined as BMI ≥ 25 (kg/m^2^), based on the cutoff point for Asian populations [14]. This study was conducted per the ethical principles stated in the Declaration of Helsinki and was approved by the Independent Ethics Committee of Saitama Medical University International Medical Center.

### Comparisons of LDG and RDG in the correlations between BMI and surgical outcomes

Correlations between BMI and operating time, as well as BMI and blood loss, were investigated separately for the LDG and RDG groups. The correlation between BMI and CRP levels on postoperative day (POD) 3 was investigated in the cohorts of all patients, as well as LDG and RDG. CRP levels on POD 3 were compared between LDG and RDG in all patients and those with BMI ≥ 25. A multivariate analysis of the factors related to CRP on POD 3 was subsequently performed in the cohort of patients with BMI ≥ 25.

### Statistical analysis

Chi-square and Fisher’s exact tests were used to analyze categorical variables, while the Mann–Whitney U test was used for continuous variables. Uni- and multi-variate analyses were performed to investigate associated factors of CRP on POD 3 in patients with BMI ≥ 25. Variables having *p* values < 0.10 in the univariate analysis were included as covariates in the multivariate analyses. Differences were considered statistically significant at *p* < 0.05. EZR, a modified version of the R Commander designed to add statistical functions, was used to perform all statistical analyses.

## Results

### Patients’ characteristics

**Fig. 1:**
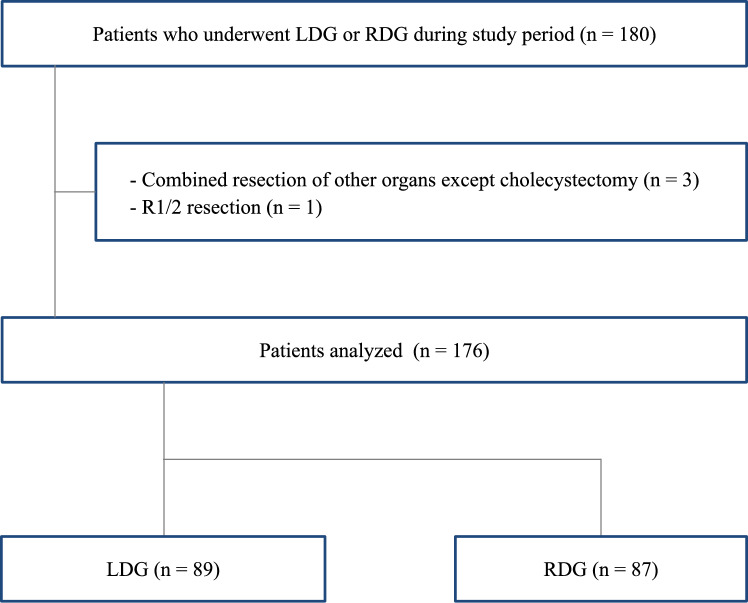
Flowchart of patient selection. Among the 180 patients who underwent laparoscopic distal gastrectomy (LDG) or robot-assisted distal gastrectomy (RDG), 176 patients, excluding 4 who met the exclusion criteria, were included in the analysis. The number of patients in the LDG and RDG groups was 89 and 87, respectively

Overall, 180 patients who underwent LDG or RDG during the study period were retrospectively reviewed. Among them, three patients who underwent combined resection of organs other than cholecystectomy and one patient who underwent noncurative resection were excluded from the analysis. The analyzed patients were classified into LDG (*n* = 89) and RDG (*n* = 87) groups (Fig. 1). The characteristics of the overall cohort and the cohort of patients with BMI ≥ 25 are shown in Tables 1 and 2. The overall cohort included 116 males and 60 females, with a mean age of 73 years (interquartile range [IQR], 68–78 years). The median BMI was 22.4. The numbers of patients with pStages I, II, III, and IV were 108 (61.4%), 39 (22.2%), 29 (16.5%), and 0 (0.0%), respectively. The LDG group had significantly more males and cases of gastric cancer with pStage III compared with the RDG group. In terms of surgical outcomes, while the operating time was significantly longer for RDG (median: 284 min and 355 min in LDG and RDG, respectively), no statistical differences were observed in blood loss or postoperative complications with Clavien–Dindo (CD) ≥ 2. Second, in the cohort of patients with BMI ≥ 25, the number of patients was 38, including 19 in LDG and 20 in RDG. This cohort comprised 28 males and 10 females, with a mean age of 72.5 years (IQR: 65.25–79.5). When focusing on the patients with BMI ≥ 25, the differences in the rates of male/female and pStages disappeared. Although a statistical difference in operating time remained, the median operating time of LDG approached that of RDG (312 min and 356 min for LDG and RDG, respectively). In particular, the median operating time of RDG was almost the same between all patients and patients with BMI ≥ 25. Blood loss and postoperative complications with CD ≥ 2 did not show the statistical differences between LDG and RDG as with the cohort of all patients.

**Table 1:** Patient characteristics

	Total (*n* = 176)	LDG (*n* = 89)	RDG (*n* = 87)	*p*-value
Age (years, median [IQR])	73 (68–78)	73 (65–78)	73 (70–78)	0.209
Sex				
Male	116 (65.9%)	49 (55.1%)	67 (77.0%)	0.003
Female	60 (34.1%)	40 (44.9%)	20 (23.0%)	
BMI (kg/m^2^, median [IQR])	22.4 (20.8–24.7)	22.4 (20.8–24.5)	22.4 (20.9–24.8)	0.686
Lymph node dissection				
D1	2 (1.1%)	2 (2.2%)	0 (0.0%)	0.614
D1+	127 (72.2%)	63 (70.8%)	64 (73.6%)	
D2	47 (26.7%)	24 (27.0%)	23 (26.4%)	
Reconstruction				
Billroth-I	111 (63.1%)	56 (62.9%)	55 (63.2%)	0.862
Billroth-II	26 (14.8%)	12 (13.5%)	14 (16.1%)	
Roux-en-Y	39 (22.2%)	21 (23.6%)	18 (20.7%)	
Operating time (min, median [IQR])	324 (277.5–369)	284 (246–328)	355 (323.5–391.5)	< 0.001
Blood loss (mL, median [IQR])	10 (0–20)	1 (0–23)	10 (0–20)	0.631
Postoperative complications with CD grade ≥ 2	21 (11.9%)	9 (10.1%)	12 (13.8%)	0.493
Histological type				
tub1	37 (21.0%)	14 (15.7%)	23 (26.4%)	0.102
tub2	48 (27.3%)	25 (28.1%)	23 (26.4%)	
pap	3 (1.7%)	3 (3.4%)	0 (0.0%)	
por	69 (39.2%)	39 (43.8%)	30 (34.5%)	
sig	7 (4.0%)	5 (5.6%)	2 (2.3%)	
muc	4 (2.3%)	1 (1.1%)	3 (3.4%)	
Others	8 (4.6%)	2 (2.2%)	6 (6.9%)	
pStage				
I	108 (61.4%)	56 (62.9%)	52 (59.8%)	0.049
II	39 (22.2%)	14 (15.7%)	25 (28.7%)	
III	29 (16.5%)	19 (21.3%)	10 (11.5%)	
IV	0 (0.0%)	0 (0.0%)	0 (0.0%)	

LDG, laparoscopic distal gastrectomy; RDG, robot-assisted distal gastrectomy; IQR, interquartile range; BMI, body mass index; CRP, C-reactive protein; CD, Clavien–Dindo

**Table 2:** Patient characteristics in the group with BMI ≥ 25

	Total (*n* = 38)	LDG (*n* = 18)	RDG (*n* = 20)	*p*-value
Age (years, median [IQR])	72.5 (65.25–79.5)	72 (62.25–81)	72.5 (67–77.25)	0.838
Sex				
Male	28 (73.7%)	13 (72.2%)	15 (75.0%)	1.000
Female	10 (26.3%)	5 (27.8%)	5 (25.0%)	
BMI (kg/m^2^, median [IQR])	26.9 (26.2–29.4)	27.4 (26.4–29.4)	26.7 (26.1–28.8)	0.335
Lymph node dissection				
D1	1 (2.6%)	1 (5.6%)	0 (0.0%)	0.838
D1+	30 (78.9%)	14 (77.8%)	16 (80.0%)	
D2	7 (18.4%)	3 (16.7%)	4 (20.0%)	
Reconstruction				
Billroth-I	21 (55.3%)	8 (44.4%)	13 (65.0%)	0.509
Billroth-II	9 (23.7%)	5 (27.8%)	4 (20.0%)	
Roux-en-Y	8 (21.1%)	5 (27.8%)	3 (15.0%)	
Operating time (min, median [IQR])	333.5 (304.75–376.5	312 (279–352)	356 (325.25–391.25)	0.003
Blood loss (mL, median [IQR])	12.5 (0–32.25)	20 (0–46.25)	10 (0–30)	0.346
Postoperative complications with CD grade ≥ 2	1 (2.6%)	1 (5.6%)	1 (5.0%)	1.000
Histological type				
tub1	5 (13.2%)	2 (11.1%)	3 (15.0%)	0.879
tub2	12 (31.6%)	7 (38.9%)	5 (25.0%)	
pap	0 (0.0%)	0 (0.0%)	0 (0.0%)	
por	18 (47.4%)	8 (44.4%)	10 (50.0%)	
sig	2 (5.3%)	1 (5.6%)	1 (5.0%)	
muc	1 (2.6%)	0 (0.0%)	1 (5.0%)	
Others	0 (0.0%)	0 (0.0%)	0 (0.0%)	
pStage				
I	27 (71.1%)	13 (72.2%)	14 (70.0%)	0.396
II	7 (18.4%)	2 (11.1%)	5 (25.0%)	
III	4 (10.5%)	3 (16.7%)	1 (5.0%)	
IV	0 (0.0%)	0 (0.0%)	0 (0.0%)	

LDG, laparoscopic distal gastrectomy; RDG, robot-assisted distal gastrectomy; IQR, interquartile range; BMI, body mass index; CRP, C-reactive protein; CD, Clavien–Dindo

### Correlations between BMI and operating time/blood loss

**Fig. 2:**
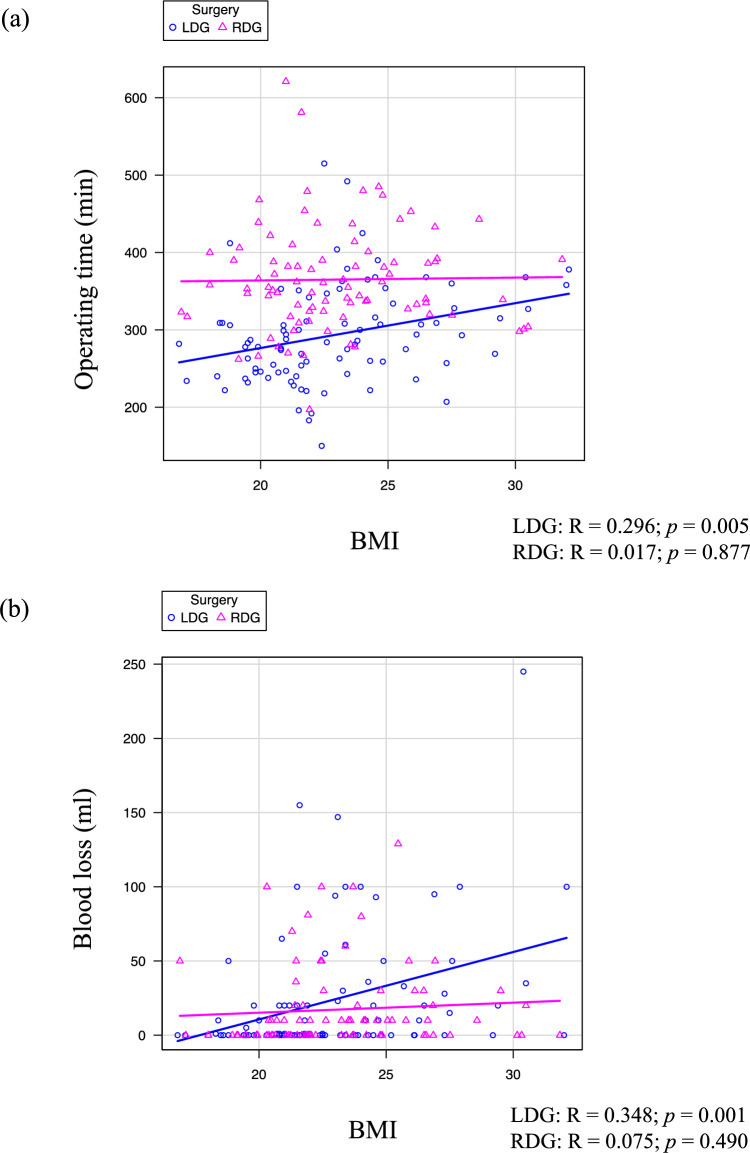
Correlation between body mass index (BMI) and surgical outcomes. The relationships between BMI, operating time (**a**), and blood loss (**b**) were investigated. Scatter plots were divided into laparoscopic distal gastrectomy (LDG) and robot-assisted distal gastrectomy (RDG) groups (LDG: *R* = 0.296, *p* = 0.005 and RDG: *R* = 0.017, *p* = 0.877 in operating time; LDG: *R* = 0.348, *p* = 0.001 and RDG: *R* = 0.075, *p* = 0.490 in blood loss).

The correlations between BMI and operating time were investigated separately for LDG and RDG (Fig. 2a). While BMI in the LDG group was significantly correlated with operating time, BMI in the RDG group did not correlate (LDG: *R* = 0.296, *p* = 0.005; RDG: *R* = 0.017, *p* = 0.877). Similarly, the correlation between BMI and blood loss was investigated separately for LDG and RDG (Fig. 2b). While BMI in the LDG group was significantly correlated with blood loss, BMI in the RDG group was not correlated with operating time (LDG: *R* = 0.348; *p* = 0.001, RDG: *R* = 0.075; *p* = 0.490).

### Comparison of the association of BMI to CRP on POD 3 between LDG and RDG

**Fig.3:**
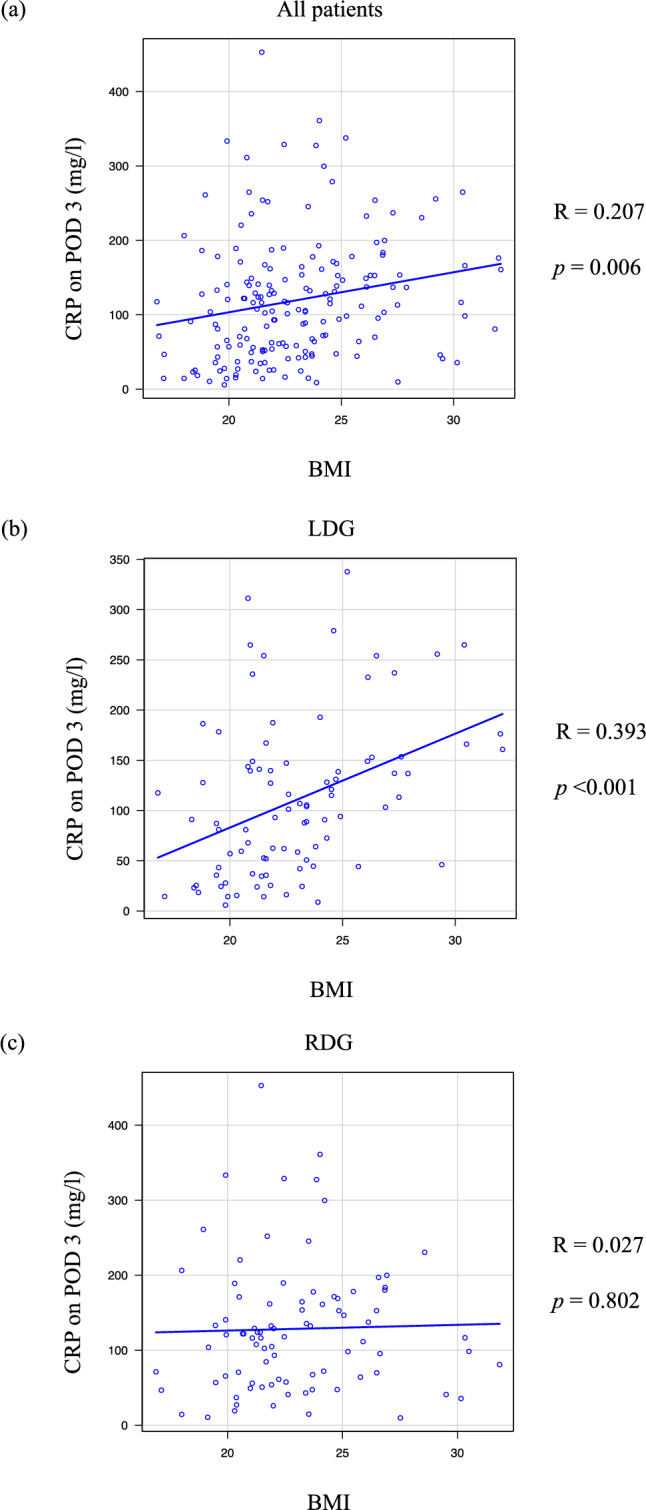
Correlation between body mass index (BMI) and C-reactive protein (CRP) level on postoperative day (POD) 3. Correlations between BMI and CRP on POD3 were investigated in all included patients (*R* = 0.207, *p* = 0.006) (**a**), patients who underwent laparoscopic distal gastrectomy (LDG) (*R* = 0.393, *p* < 0.001) (**b**), and patients who underwent robot-assisted distal gastrectomy (RDG) (*R* = 0.027, *p* = 0.802) (**c**)

**Fig. 4:**
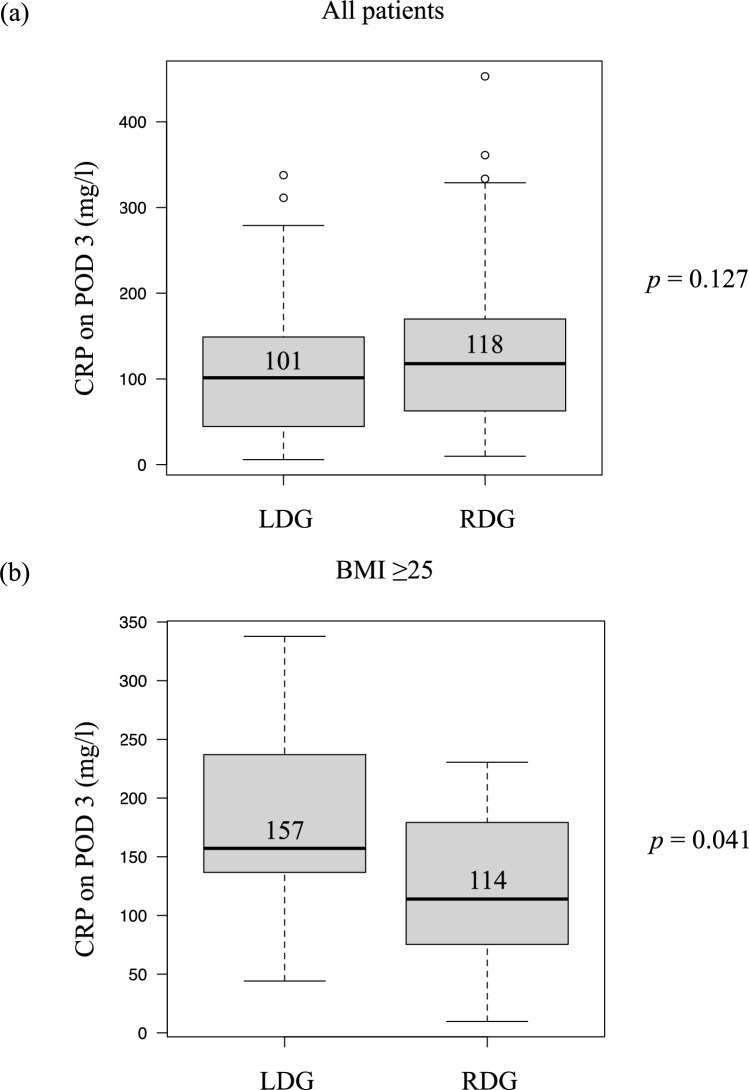
Comparison of C-reactive protein (CRP) level on postoperative day (POD) 3. Comparisons of CRP on POD3 between laparoscopic distal gastrectomy (LDG) and robot-assisted distal gastrectomy (RDG) were performed in all included patients (**a**) and the patients with BMI ≥ 25 kg/m^2^ (**b**)

Correlations between BMI and CRP levels on POD 3 were investigated in all included patients in the LDG and RDG groups (Fig. 3). Analysis of all patients showed a statistically significant correlation between BMI and CRP levels on POD 3 (*R* = 0.207; *p* = 0.006). While the correlation became stronger in the LDG analysis, the RDG analysis did not show a correlation between BMI and CRP on POD 3 (LDG: *R* = 0.393, *p* < 0.001; RDG: *R* = 0.027, *p* = 0.802). Although CRP on POD 3 did not differ between LDG and RDG in all patients, LDG showed significantly higher CRP on POD 3 than RDG in patients with BMI ≥ 25 (Fig. 4).

**Table 3:** Factors related to CRP level on postoperative day 3 in patients with BMI ≥ 25

	Univariate regression analysis			Multiple regression analysis		
	B	*p*-value	95% CI	B	*p*-value	95% CI
Age ≥ 70 years	2.390	0.348	− 2.710–7.490			
Male	0.227	0.935	− 5.336–5.789			
RDG	− 5.202	0.027	− 9.782–0.621	− 6.887	0.003	− 11.306 to − 2.469
D2 lymph node dissection	3.814	0.213	− 2.297–9.925			
Operating time ≥ 360 min	4.161	0.081	− 0.539–8.861	6.068	0.011	1.495–10.642
Blood loss ≥ 50 mL	2.227	0.454	− 3.734–8.188			
Postoperative complications with CD grade ≥ 2	6.630	0.219	− 4.109–17.370			
pStage ≥ 2	1.564	0.559	− 3.811–6.940			

CRP, C-reactive protein; BMI, body mass index; CI, confidence interval; RDG, robot-assisted distal gastrectomy; CD, Clavien–Dindo

### Factors related to elevation of CRP on POD 3 in patients with BMI ≥ 25

Univariate and multivariate analyses for CRP on POD 3 were performed in the cohort of patients with BMI ≥ 25. The univariate analysis suggested that RDG and operating time ≥ 360 min were associated with CRP elevation. In a multivariate analysis including these two factors as covariates, both two factors demonstrated as independent related factors of CRP level on POD 3 (*B* = − 0.6887; *p* = 0.003 and *B* = 6.068; *p* = 0.011 in RDG and operating time ≥ 360, respectively) (Table 3).

## Discussion

Gastrectomy for the obese patients with gastric cancer is technically challenging, primarily due to the large amount of intra-abdominal adipose tissue. We previously reported the clinical advantages of LDG over open distal gastrectomy in patients with obesity [15]. A magnified view provided by a laparoscope, precise manipulation with delicate forceps, and a smaller incision compared with open surgery enable accurate and minimally invasive gastrectomy. In addition to these advantages, robotic surgery offers a highly immersive magnified 3D view and enhanced ergonomics through the functionality of diarthrodial joints [16–18].

Our findings indicate that while the operating time for RDG is generally longer than that for LDG, it did not increase in patients with a high BMI, whereas the operating time for LDG increased with BMI. Similarly, blood loss in LDG increases with BMI, whereas blood loss in RDG does not correlate with BMI. Consequently, the trend of blood loss volume in LDG and RDG is reversed between patients with low and high BMI, suggesting a clinical advantage of RDG in reducing blood loss in patients with higher BMI. Previous studies have shown that RG reduces blood loss despite a longer operating time compared with LG [19]. Our study further suggests the advantage of RG in reducing blood loss, particularly in patients with high BMI.

As mentioned earlier, our previous study identified a correlation between BMI and CRP levels after gastrectomy in patients who underwent LDG [9]. This study demonstrated that BMI had a significant correlation with CRP on POD 3 in LDG, consistent with our previous findings; however, RDG showed no such correlation. These findings suggest that RDG stabilizes postoperative inflammation regardless of BMI. Several reports have indicated that RG does not provide an advantage in terms of postoperative inflammatory markers, including CRP, compared with LG [20, 21]. However, our findings suggest that the true benefit of RG in mitigating postoperative inflammation is particularly evident in patients with obesity. The clinical benefit of robotic surgery in reducing postoperative surgical stress has been demonstrated in colorectal cancer surgeries [22, 23]. Multivariate analysis identified LDG, compared with RDG, as an independent risk factor for elevated CRP levels on POD 3 in patients with BMI ≥ 25. Conversely, a longer operating time (≥ 360 min) was also suggested as a risk factor for postoperative CRP elevation. Although the longer operating time of RG is generally considered a disadvantage, our study showed that the difference in operating time between LG and RG diminished as BMI increased. This observation further supports the finding that RDG suppressed the postoperative inflammatory status only in patients with a high BMI compared with LDG.

The previous studies have reported that visceral fat volume is significantly related to postoperative CRP levels and that visceral obesity exacerbates the postoperative inflammatory response [24, 25]. RG, with its highly immersive magnified 3D view, aids in identifying the boundaries of adipose tissue and allows for precise movement using diarthrodial joints, minimizing damage to surrounding organs even in cases of poor visibility caused by excessive intra-abdominal fat. Moreover, a previous report suggested the surgical benefit of RG using bipolar forceps compared to laparoscopic devices in terms of thermal damage to nearby organs [26]. The other report indicated the reduced incidence of pancreatic fistula in RG compared with LG as one of the surgical benefits of RG [27]. Although clarifying the detailed mechanisms could be difficult, robotic surgery mainly using bipolar forceps has possibility to be well suited to surgical procedures of gastrectomy, that involve peeling off layers of tissues or organs. High postoperative inflammatory status has been reported as a risk factor for long-term prognosis in gastric cancer surgery [10, 28]; therefore, performing gastrectomy with suppression of elevated postoperative inflammatory status is important. Our findings suggest additional clinical advantages of RG, particularly in patients with obesity.

This study has several limitations. First, the study defined patients with obesity as those with BMI ≥ 25; however, in Western countries, severe obesity (BMI ≥ 30 or ≥ 35) is more prevalent. Our data included only eight patients with BMI ≥ 30. Thus, our findings in Japanese patients may not directly apply to those in Western countries. Further investigations in the robotic surgery for the patients with severe obesity are expected. Second, this study exclusively analyzed patients who underwent DG, making it difficult to generalize the findings to those who underwent total or proximal gastrectomy. Third, RDG was a relatively new surgical procedure and required different skills than traditional laparoscopic surgery, meaning our surgical team may have been on a learning curve of robotic surgery even after excluding the cases in the early implementation period of RG from the analysis [29]. However, this limitation likely underestimates the outcomes of RDG, suggesting that further improvements can be expected as surgeons gain more experience. Fourth, the risk of bias cannot be excluded due to the single-center, retrospective design of the study. We reviewed all patients who underwent distal gastrectomy during the study period and analyzed the data from all patients who met the inclusion criteria to minimize bias. Despite these limitations, our findings provide valuable insights into the clinical advantages of RG in patients with obesity.

In conclusion, our findings suggest that RDG resulted in lower CRP on POD 3 compared with LDG in patients with BMI ≥ 25. LDG, compared with RDG, was identified as an independent risk factor for elevated CRP levels on POD 3 in patients with obesity. Although a longer operating time was also a risk factor for postoperative CRP elevation, the operating time of LDG approached that of RDG as BMI increased, while blood loss volume in LDG tended to be higher than in RDG in the obese patients. RDG was suggested to have clinical advantages compared with LDG, particularly in patients with obesity with BMI ≥ 25.

## Data Availability

No datasets were generated or analysed during the current study.
